# Relation of chelation regimes to cardiac mortality and morbidity in patients with thalassaemia major: an observational study from a large Greek Unit

**DOI:** 10.1111/j.1600-0609.2010.01491.x

**Published:** 2010-10

**Authors:** Vassilios Ladis, Giorgos Chouliaras, Vasilios Berdoukas, Panagiotis Moraitis, Kirykos Zannikos, Eleni Berdoussi, Christos Kattamis

**Affiliations:** Thalassaemia Unit, 1st Department of Paediatrics, University of Athens, ‘Aghia Sophia’ Children’s HospitalAthens, Greece

**Keywords:** thalassaemia major, cardiac disease, desferrioxamine, deferiprone, deferasirox, transfusional iron overload

## Abstract

**Objectives:**

Cardiac complications because of transfusional iron overload are the main cause of death in thalassaemia major. New chelators and iron monitoring methods such as cardiac magnetic resonance (CMR) became available after the year 2000. We evaluated the impact of these new management options on cardiac mortality and morbidity.

**Methods:**

The risk of cardiac death during 1990–1999 and 2000–2008 was compared. Furthermore, after 1999, morbidity, mortality and reversal of heart failure were evaluated according to chelation regime: desferrioxamine (DFO), deferiprone (DFP) and combination therapy of DFO and DFP. We also present preliminary results for deferasirox (DFX), a new oral chelator.

**Results:**

Three hundred and fifty-four patients were included in the *de novo* cardiac event evaluation, while 86 were included in the improvement component. The annual risk of cardiac death in patients aged between 20–30 and 30–40 reduced from 1.52% to 0.67% and 1.87% to 0.56%, respectively, before and after the year 2000. The risk for a *de novo* cardiac event for DFO was 9.1 times greater than that of DFP and 23.6 than with the combination of DFP and DFO. For DFX, there was one cardiac event over 269 patient-years. The risk of cardiac death was 9.5 per 1000 patient-years for DFO, 2.5 on DFP, 1.4 on combination. In the DFX group no cardiac deaths were recorded. The odds of improvement were 8.5 times greater with DFP and 6.1 with combination therapy compared to DFO.

**Conclusions:**

The new chelation regimes, together with CMR have contributed significantly to the reduction in cardiac morbidity and mortality in patients with thalassaemia major.

In patients with transfusion-dependent beta thalassaemia major, transfusions and iron chelation therapy with desferrioxamine (DFO) have significantly improved the survival, ameliorated clinical features and reduced morbidity (1–4). However, cardiac complications are still responsible for significant morbidity and remain the leading cause of mortality (2). In some cases, these were because of difficulty in accepting the chelation treatment, which was cumbersome (5), but also occurred even in some patients who accepted the chelation therapy well (6, 7).

Effectiveness of chelation is progressively improving, especially during the last decade, with the introduction of two oral chelators, deferiprone (DFP) and deferasirox (DFX). There are now more choices with respect to the provision of iron chelation therapy. Each chelator can be given as monotherapy and DFP and DFO have been administered widely in combination.

Since 2000, a number of observational studies have indicated an improvement in overall survival with reduction of deaths caused by cardiac disease (8, 9). In addition, two studies demonstrated that the incidence of cardiac disease and cardiac-related death were higher in patients who continued treatment with DFO compared to those who switched to DFP (10, 11). In addition to the broader therapeutic choices, newer assessment techniques for monitoring iron concentration have been developed and provide a means of accurately assessing iron overload in various organs. In particular, magnetic resonance imaging (MRI) has allowed more accurate assessment of liver and myocardial iron overload.

The initial objective of this observational study was to determine whether the availability of new treatment regimes and the ability to determine organ-specific iron load have had an impact on survival. The new treatment regimes include the availability of oral iron chelators. The organ-specific assessment of iron load is now available, particularly for the heart by T2* estimation by cardiac magnetic resonance (CMR) (12). We therefore calculated and compared the risk of cardiac death over two distinct time periods: 1990–1999 and 2000–2008. During the first time period, DFO was the only chelator available in our unit, while after 2000 other chelation options, mainly DFP and combination therapy with DFP and DFO (Combination), became available for thalassaemia patients. The next step was to analyse and compare cardiac morbidity and mortality as well as improvement of existing cardiac disease in patients receiving DFO, DFP and Combination after the year 2000. We also sought to analyse other factors that influence the incidence of cardiac events or reversal of heart disease including age, sex and severity of haemosiderosis.

With respect to DFX, a limited amount of data has been available to date as it was licensed in Europe <3 yr ago. For this reason, we did not include DFX in a comparison analysis but rather reported preliminary results in preventing and reversing cardiac morbidity and possibly mortality.

## Patients and methods

In our unit, currently, more than 400 patients with thalassaemia are treated with regular blood transfusions at 15–21 d intervals, chelation therapy and appropriate monitoring. Pretransfusion haemoglobin (Hb) levels are maintained above 95 g/L. Chelation regimes include DFO at doses of 20–40 mg/kg per infusion with at least three (3–5) infusions per week, DFP at 75–100 mg/kg per day in three divided doses, combinations of the two with DFP daily and DFO with similar doses as with monotherapy, 8–24 hourly infusions/3–7 times per week. After 2007, when DFX was licensed, it was also given in our unit as monotherapy at doses of 15–30 mg/kg per day. In some selected cases, DFX was also administered with doses of up to 40 mg/kg per day.

Iron load is traditionally assessed by regular measurement of ferritin levels. Over the last 8 years cardiac and hepatic MRI has been increasingly utilised to evaluate, indirectly, the degree of cardiac and hepatic iron loading. In general patients are assessed annually for cardiac function by symptomatology, clinical examination, chest X-rays, ECGs and echocardiography. Some patients were reluctant to have these assessments.

The decisions for the chelation regime and the doses of the drugs prescribed were based on parameters reflecting iron overload (2, 12, 13), cardiac status and other iron-related morbidities. We prescribed combination therapy in patients in whom we had a high level of concern with respect to their iron load, i.e. very high ferritin levels, liver iron concentration (LIC) or cardiac iron as shown by MRI and in patients with obvious cardiac dysfunction. We prescribed the maximum doses and frequency of use of the chelators for those patients with cardiac dysfunction. Individual patients’ personal preferences were also taken into consideration.

As cardiac morbidity and mortality (4, 14) in patients with thalassaemia have been linked to age, gender and severity of iron load, demographic data (date of birth and gender) and annual mean ferritin levels were recorded. Patients were classified according to their mean ferritin levels for each calendar year into three groups as follows: mild haemosiderosis: <2000 μg/L, moderate: 2000–4000 μg/L and severe: >4000 μg/L. Three age groups were also formed: 10–20, 20–30 and >30 yr.

For the comparison of the risk of cardiac death between 1990–1999 and 2000–2008, all patients with thalassaemia were included regardless of the chelation regime. The risk of cardiac death was evaluated in two age groups for each time period; those aged 20–30 yr and those 30–40 yr. This stratification was applied to compare similar age groups over the two time periods and to eliminate possible bias because of including patients less prone to iron-related cardiac death in the second time period.

Cardiac disease is rare before the age of 10 yr. Therefore, for the assessment of the incidence of *de novo* cardiac events according to the chelation regime, all patients with thalassaemia older than 10 yr and free of cardiac disease on 1 January 1999, who were on DFO, DFP or Combination, were included. Those who reached the 10th year of age after that date were subsequently included when reaching that age. The year 1999 was chosen because it was the last during which all patients in the study exclusively received DFO. After 2000 most patients continued on DFO including those with severe cardiac iron loading. Subsequently, progressively more patients with severe iron load were preferentially prescribed Combination or DFP. In this way, during the last 10 yr, a sufficient number of patients from each group of severity participated in those three treatment groups. DFX has been used in our unit since 2003, initially in clinical trials and later as optional monotherapy, mainly in patients with mild iron load according to ferritin levels. This policy could introduce a systematic bias as this group of patients would selectively consist of milder cases than those patients included in the other three regimes. For this reason, patients on DFX were excluded from the comparative analysis. The end point of the *de novo* cardiac event analysis was the development of a cardiac event or the 31 December 2008, if no cardiac event occurred. In this document, the terms ‘cardiac disease’, ‘cardiac dysfunction’ and ‘cardiac event’ are used interchangeably.

A *de novo* cardiac event was defined as either of the following:

- Prescription of cardiac medication by cardiologists for clinically evident heart failure, after they had fully assessed their cardiac status as described earlier.
- Cardiac death without pre-existing evidence of heart disease.

Cardiac medications included angiotensin-converting enzyme inhibitors, angiotensin receptor blockers, digoxin and beta-blockers. Diuretics were considered as cardiac medication only when given in conjunction with other cardiac medications. In thalassaemia, some patients are treated with diuretics for the management of hypercalcuria and not as a cardiac medication.

The analyses were conducted according to single calendar year intervals between 1 January 1999 and 31 December 2008. Our main approach was to evaluate the probability (risk) of developing a cardiac event for each chelation regime in each calendar year. This risk was calculated by dividing the number of new cardiac events in each regime by the total number of patients attributed to that regime for each calendar year. Each patient was included as receiving one particular regime during each year. In switching between regimes, the following rules were imposed when attributing patients to a particular group: For each calendar year, the patients were assigned to the particular treatment regime they received for the longest period provided that it lasted for at least 6 months of that year. On this basis, 18 patients were excluded, none of whom developed a cardiac event during this period. On identification of a cardiac event, this was attributed to the regime the patient was receiving for the longest period during the previous 12 months. For the estimation of exact incidence rates for each regime, the events were also analysed according to the regime received at the time of each event.

The risk of cardiac death in relation to the chelation regime was also evaluated. Each cardiac death was attributed to the regime the patient was receiving for the longest period during the 12 months before death.

All patients who developed a *de novo* cardiac event after 1999 as well as those with evidence of cardiac disease prior to 1999 were included in the improvement component of the analysis. Exceptions were patients who experienced sudden cardiac death without evidence of pre-existing cardiac disease and those who developed a cardiac event during 2008. The latter group was excluded as less than 12 months remained until the end of the study. Reversal of cardiac disease was defined as cessation of cardiac medications after cardiological review. Similar criteria to those described for the *de novo* component, with respect to regime exposure were used for attributing the reversal.

Only descriptive data on the incidence of cardiac-related deaths, *de novo* cardiac event and reversal are reported for DFX. Compliance with the chelation therapy and dosing were not assessed or taken into account for the purposes of this study.

### Statistical analysis

Continuous variables are presented as mean ± SD while categorical variables are described using absolute and relative frequencies. For ferritin and time of exposure in survival analysis median and interquartile range (IQR) are also reported. Hypotheses on whether the mean of a continuous variable differed significantly from that of at least one other group were tested by analysis of variance (anova). Whenever multiple hypotheses testing occurred, the significance level was corrected by the Bonferroni method (0.05 divided by the total number of comparisons). Categorical variables were tested using Fisher’s exact test.

For the comparison on risk of death between the intervals 1990–1999/2000–2008 a Poisson distribution was utilised. In this case, age-related rates of death were calculated and compared according to each person’s contribution in each age-period.

In the *de novo* cardiac event analysis a 2 × 3 table (new cardiac events vs. patients at risk for each of the three treatment groups) was constructed for each calendar year and the respective probabilities (risk) along with the exact 95% confidence intervals (C.I.) were reported. Subsequently, the 10 -yr data were combined. From these combined data, the comparisons of the probabilities of experiencing an event among the three treatment groups were performed by logistic regression and the relative risks are reported as odds ratios (ORs) and 95% C.I.s. Age, gender and level of haemosiderosis were assessed as potential confounders in the final logistic regression. A small number of treatment groups either had few or no *de novo* cardiac events. For this reason it was necessary to perform an exact logistic analysis to derive ORs and 95% C.I.s (15). To report incidence rates of *de novo* cardiac events for each regime, we used a time to event method and the event was attributed to the regime the patient was taking on the date of the event.

A similar approach in which the events were compared between regimes was also used for the assessment of improvement. This included a 2 × 3 table (patients improved vs. patients with cardiac disease for each of the three treatment groups) for each calendar year and a comparison with logistic regression of the 9 yr combined data. A rate was also calculated.

To test whether the risk of dying from cardiac complication differs significantly among the three treatment groups, we applied a time to event approach with an exact Poisson regression model, to overcome the difficulty of the presence of zero events in several sub-groups. The starting point was the 1 January 1999. After that time point, whenever a patient changed regime a new record was generated and the time was measured from the start for as long as the patient remained on the new regime. In addition the mean ferritin levels during the time period of each record as well as the age at the end of each record was recorded. In this way the majority of patients contributed information to more than one regime, allowing for time variation in treatment, ferritin levels and age. The end point was the 31 December 2008 or the date of death from cardiac complications, whichever occurred first. Patients who died of non-cardiac-related causes or underwent bone marrow transplantation were considered censored observations at the time of death or the procedure.

All data were entered in a Microsoft Access database and statistical analysis was performed using stata 11.0 (StataCorp. College Station, TX, USA).

The Ethics Committee of the hospital approved permission for medical review, waiver of informed consent and anonymous publication of data according to the Declaration of Helsinki.

## Results

### Patient characteristics

For the analysis of the risk of cardiac-related deaths, 597 patients (51.6% males) were included for the time period of 1990–1999 and 527 patients (49.5% males) were included in the period 2000–2008.

For the *de novo* cardiac event component, a total of 354 patients were included, 346 as from the 1 January 1999, while eight additional patients reached the age of 10 yr during the study period and were progressively included. A total of 63 *de novo* cardiac events were recorded. Table 1 demonstrates the demographic data for all patients.

**Table 1:** Characteristics of patients included in the *de novo* cardiac event component

Treatment group, (*N*)	Age^1^, mean (SD)	Ferritin^2^, mean (SD), median (IQR)	Gender^3^: males, *n* (%)
DFO (343)	21.2 (6.9)	2500 (1653), 2014 (1322–3231)	176 (51.3)
DFP (97)	25.3 (6.8)	2903 (2003), 2453 (1297–4330)	46 (47.4)
Combination (166)	26.7 (6.0)	2551 (1795), 2007 (1206–3678)	79 (47.6)
DFX (118)	26.0 (8.7)	2673 (1765), 2076 (1284–3718)	45 (38.1)

IQR, interquartile range; DFO, desferrioxamine; DFP, deferiprone; DFX, deferasirox.

1 anova: *F*-test = 31.3, *P* < 0.001. Significant comparisons DFP vs. DFO, Combination vs. DFO, DFX vs. DFO (all *P*-values <0.001 were statistically significant considering 0.05/6 = 0.008 as the confidence level after the Bonferroni correction).

2 anova: *F*-test = 1.4, *P* = 0.23.

3 Fisher’s exact test: *P* = 0.10.

For the improvement component, 86 persons were included in the analysis. This group consisted of the following: (i) twenty-nine patients who had a history of cardiac disease before 1999 and were not included in the *de novo* event component. (ii) Two patients who developed a cardiac event during the study period but were not included in the *de novo* component as they had no cardiac assessments until the diagnosis of their heart decompensation. (iii) From the 63 new events, 55 were included in the improvement component. One was excluded as his cardiac event occurred during 2008, while seven patients, who died suddenly from cardiac causes and who had no evidence of pre-existing cardiac disease, were not included. Demographic data of the patients evaluated in the improvement component in all regimes are shown in Table 2.

**Table 2:** Characteristics of patients included in the improvement component

Treatment group, (*N*)	Age^1^, mean (SD)	Ferritin^2^, mean (SD), median (IQR)	Gender^3^: males, *n* (%)
DFO (75)	26.8 (5.7)	2956 (2222), 2511 (1258–4302)	51 (68.0)
DFP (35)	31.0 (6.7)	2252 (2573), 1151 (407–3025)	21 (60.0)
Combination (61)	30.1 (6.2)	2927 (2386), 2135 (1023–3912)	36 (59.0)
DFX (9)	33.9 (6.2)	2332 (1959), 1483 (1155–3009)	5 (55.6)

IQR, interquartile range; DFO, desferrioxamine; DFP, deferiprone; DFX, deferasirox.

1 anova: *F*-test = 8, *P* < 0.001. Significant comparisons (considering 0.05/6 = 0.008 as the confidence level after the Bonferroni correction): DFP vs. DFO (*P*-value = 0.002), Combination vs. DFO (*P*-value = 0.004), DFX vs. DFO (*P*-value = 0.006).

2 anova: *F*-test = 1, *P* = 0.37.

3 Fisher’s exact test: *P* = 0.65.

For each treatment group, the data in Tables 1 and 2 were derived from all the individuals assigned to the particular regime during the observational period. Thus, a patient might contribute information to more than one of the three regimes. For this reason, the sum of patients in the four groups was greater than the total number of patients in this study.

### Trends in cardiac deaths

**Figure 1:**
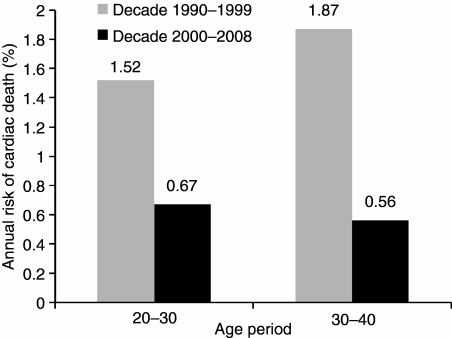
Comparison of annual risk of cardiac death of the years 1990–1999 compared to 2000–2008 stratified according to age.

Table 3 and Fig. 1 show the risk of cardiac-related death during 1990–1999 compared to 2000–2008 for the two age groups of 20–30 and 30–40 yr. There was an approximately threefold significant reduction in the second time interval (2000–2008) in both age groups.

**Table 3:** Age-specific risk of cardiac death according to decade

	1990–1999			2000–2008			
Age group (yrs)	Deaths	Person-years	Risk (per 1000 person-years)	Deaths	Person-years	Risk (per 1000 person-years)	Risk ratio (*P*)
20–30	30	1964.5	15.2	14	2079.7	6.7	2.2 (0.0095)
30–40	5	267.5	18.7	8	1415.1	5.6	3.3 (0.052)

### *De novo* cardiac events

Sixty-three (63) *de novo* cardiac events were recorded. Table 4 shows the number of patients on DFO, DFP and Combination in chronological annual order, as well as the risk of *de novo* cardiac event for each treatment group. Logistic regression analysis (Table 5) of the 10 -yr combined data showed that patients on DFP and Combination had 9.1 times and 23.6 times, respectively, lower risk of *de novo* cardiac event compared to DFO. Patients on any DFP (DFP or Combination) showed 14 times lower risk compared to the DFO group (95% C.I. 4.3–45.4, *P* < 0.001). There was no significant difference between the risk in patients on DFP and Combination. These results are adjusted for age, gender and level of haemosiderosis that were shown to be significant confounding variables in the final regression model.

**Table 5:** Comparison^1^ of risk of *de novo* cardiac event of combined 10 yr data

Variable	Comparison	Odds ratio	95% C.I.	*P*-value
Treatment^2^	DFO vs. DFP	9.1	2.2–38.3	0.003
	DFO vs. comb	23.6	3.2–172.1	0.002
Age group	20–30 yr vs. 10–20 yr	1.9	0.9–3.9	0.073
	>30 yr vs. 10–20 yr	1.4	0.6–3.7	0.45
	20–30 yr vs. >30 yr	1.3	0.6–2.9	0.48
Gender	Males vs. females	2.7	1.5–4.9	0.001
Haemosiderosis	Moderate vs. mild	1.8	0.9–3.5	0.064
	Severe vs. mild	4.1	2.1–7.7	<0.001
	Severe vs. moderate	2.2	1.2–4.2	0.014

DFO, desferrioxamine; DFP, deferiprone.

1 Logistic regression, the reported odds ratios are adjusted for the presence of the other variables.

2 The other comparisons between the treatment groups were not significant.

**Table 4:** Annual risk of *de novo* cardiac event according to the chelation regime

	DFO	DFP	Comb
Year	Events/exposed^1^, % risk (95% C.I.)	Events/exposed, % risk (95% C.I.)	Events/exposed, % risk (95% C.I.)
1999	16/338 4.7 (2.7–7.6)	0/0 (n.a.)	0/0 (n.a.)
2000	8/316 2.5 (1.1–4.9)	0/2 0 (0–84.1)^2^	0/4 0 (0–60.2)^2^
2001	13/282 4.6 (2.5–7.7)	0/16 0 (0–20.6)^2^	0/17 0 (0–19.5)^2^
2002	7/246 2.8 (1.2–5.4)	0/18 0 (0–18.5)^2^	0/40 0 (0–8.8)^2^
2003	5/189 2.6 (0.9–6.1)	1/42 2.4 (0.06–12.6)	0/63 0 (0–5.7)^2^
2004	6/147 4.1 (1.5–8.7)	0/63 0 (0–5.7)^2^	0/69 0 (0–5.2)^2^
2005	1/111 0.9 (0.02–4.9)	1/58 1.7 (0.04–9.2)	0/92 0 (0–3.9)^2^
2006	2/69 2.9 (0.4–10)	0/50 0 (0–7.1)^2^	1/115 0.9 (0.02–4.7)
2007	1/43 2.3 (0.06–12.3)	0/35 0 (0–10)^2^	0/99 0 (0–3.6)^2^
2008	0/33 0 (0–10.6)^2^	0/43 0 (0–8.2)^2^	0/98 0 (0–3.6)^2^

DFO, desferrioxamine; DFP, deferiprone.

1 Number of persons at risk for *de novo* cardiac event in each treatment group.

2 One-sided, 97.5% confidence interval.

**Figure 2:**
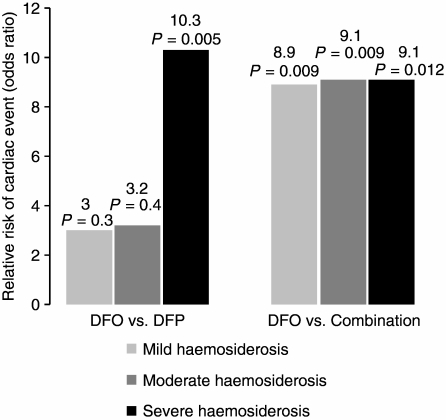
Relative risk (odds ratio) of development of *de novo* cardiac event – DFO compared to DFP and Comb, within each stratum of haemosiderosis, as estimated by exact logistic regression analysis. Numbers on the top of each bar are the odds ratio and the respective *P*-value (results adjusted for sex and age).

Figure 2 shows the odds ratio of the development of a *de novo* cardiac event of DFO compared to the other two regimes within each grade of haemosiderosis. For Combination, the effect is similar in all levels of iron load while for DFP the protective effect is more prominent for patients with severe iron load.

The rates of events according to the regime the patients were taking at the time of onset of the event were 3.3 per 100 person-years for DFO (95% C.I. 2.5–4.2, 58 events median time on DFO = 2.4 yr, IQR = 1–4.2 yr), 0.3 per 100 person-years for DFP (95% C.I. 0.04–2.2, one event recorded in a patient after 4 yr on DFP), 0.6 per 100 person-years for Combination (95% C.I. 0.2–1.7, three events recorded in three patients who had been for 6 months, 73 and 35 d, respectively, on combination therapy) and 0.4 for DFX (95% C.I. 0.05–2.6). For DFΧ 1 cardiac event was observed over 269 person-years and the patient who experienced the event was 15 months on DFX.

### Improvement component

In all patients with cardiac improvement and a large number of patients who have not been included as an improvement, the left ventricular ejection fraction increased to normal levels (>60%) but they were only included as an improvement once the cardiologist had recommended cessation of cardiac medications. Table 6 shows the analysis of the improvement according to single calendar year intervals. Logistic regression analysis of the 9 -yr combined data showed that patients on DFP were 8.5 times more likely to improve than those on DFO (OR = 8.5, 95% C.I.: 2.2–32.9, *P*-value = 0.002) while those on Combination had a 6.1 time greater likelihood (OR = 6.1, 95% C.I.: 1.7–21.2 *P*-value = 0.005). The patients on any DFP (DFP or Combination) had a 6.7 times greater chance of improving compared to DFO (OR = 6.7, 95% C.I.: 2–22.7 *P*-value = 0.002). The comparison between DFP and Combination was not significant. Age and gender were not significant while increasing level of haemosiderosis reduced the likelihood of improvement (results available from authors).

**Table 6:** Annual probability of improvement according to the chelation regime. Irrespective of the regime to which the event was attributed, patients subsequently may have changed regime explaining the increasing number of patients in the non-DFO regimes compared to the number of events that were attributed to those regimes

	DFO	DFP	Comb
Year	Improvements/exposed^1^ % (95% C.I.)	Improvements/exposed % (95% C.I.)	Improvements/exposed % (95% C.I.)
2000	2/39 5.1 (0.6–17.3)	0/1 0 (0–97.5)^2^	0/0 (n.a.)
2001	1/42 2.4 (0.06–12.6)	0/1 0 (0–97.5)^2^	0/1 0 (0–97.5)^2^
2002	0/32 0 (0–10.9)^2^	0/6 0 (0–45.9)^2^	0/14 0 (0–20.6)^2^
2003	0/21 0 (0–16.1)^2^	1/12 8.3 (0.2–38.5)	2/24 8.3 (1–27)
2004	0/19 0 (0–17.6)^2^	1/16 6.2 (0.2–30.2)	0/24 0 (0–14.2)^2^
2005	0/11 0 (0–28.5)^2^	2/15 13.3 (1.6–40.4)	0/34 0 (0–10.3)^2^
2006	0/7 0 (0–40.9)^2^	1/14 7.1 (0.2–33.9)	3/38 7.9 (1.6–21.4)
2007	0/5 0 (0–52.2)^2^	3/11 27.3 (6–61)	7/36 19.4 (8.2–36.1)
2008	0/4 0 (0–60.2)^2^	1/9 11.1 (0.3–48.2)	5/29 17.2 (5.8–35.8)

DFO, desferrioxamine; DFP, deferiprone.

1 Number of persons with heart disease in each treatment group.

2 One-sided, 97.5% confidence interval.

The rates of improvement according to the regime the patients were taking at the time of improvement, were 0.6 per 100 person-years for DFO (95% C.I. 0.1–2.3), 13.9 per 100 person-years for DFP (95% C.I. 7.1–25.1), 6 per 100 person-years for Combination (95% C.I. 3.6–11.1). For DFX, the one patient with a cardiac event improved giving a rate of improvement of 6.6 per 100 patient-years (95% C.I. 0.9–46.8).

### Incidence of cardiac deaths

After 1999, there were 22 deaths in total over the period of observation. Twenty were cardiac-related and two were from other causes (one accident and one thromboembolic event). There were 18 cardiac-related deaths in the DFO arm over 1900 patient-years (Risk = 9.5/1000 patients-years, median time of exposure = 2.6 yr, IQR = 1.3–3.6 yr), on in the DFP group for 393 patient-years (Risk = 2.5/1000 patients-years, the one death occurred in a patient after 1.3 yr on DFP) and one in the Combination group over 709 patient-years (Risk = 1.4/1000 patients-years, the one death occurred in a patient after 4.1 yr on combination therapy). An exact Poisson regression analysis (adjusted for sex, degree of haemosiderosis – age not significant) showed the following relative risk (RR) of experiencing a cardiac-related death: DFO had 6.1 RR and 5.7 RR compared to DFP and Combination, respectively, although the results were marginally non-significant (*P*-values 0.058 and 0.073, respectively). Comparison between DFP and Combination was not significant. The RR for DFO compared to DFP and Combination patients together (i.e. any DFP) was 5.2 and the *P*-value was 0.009. There were no deaths in patients taking DFX over a total of 283 patient-years.

## Discussion

This study assessed whether new therapeutic options for the management of thalassaemia have had an impact on cardiac disease free survival in a large group of patients attending one clinic. It is obvious that the risk of cardiac disease has decreased significantly after the start of this millennium as shown by the comparison of the 1990–1999 and 2000–2008 time intervals, similar to reports from other centres (8, 9). The argument though, that perhaps all patients who were more likely to present cardiac problems have died, does not hold. Our analysis ensures that similar age groups are compared, thereby, reducing the risk of including patients more vulnerable to cardiac disease in the 1990–1999 time period. This improvement can be attributed to the greater availability of choices in chelation therapy, the ability to assess cardiac and hepatic iron by MRI, improvement in compliance associated with the knowledge of the degree of cardiac and hepatic iron and tailoring of chelation therapy. The greater ability of DFP to remove cardiac iron and increase ejection fraction (16) is likely to have played a significant role in this improvement. Its theoretical ability to reduce labile cellular iron and thereby reduce free radical activity is also likely to play a role (17). This cardio-protective effect was also shown in a recent randomised study in which patients on DFO had a 27.78 greater risk of cardiac death compared to patients receiving DFP or Combination (18). This together with our data clearly demonstrates that the decrease in the incidence of cardiac-related deaths is more prominent in patients receiving any DFP regime. The latter study concluded that the protective effect of DFP appeared irrespective of the degree of body iron load. The lower protective effect found in our study (relative risk of death 5.2) is most likely because of our policy of changing the treatment of our patients from DFO to a DFP regime, in contrast to the randomised study, once we became concerned about their cardiac function.

The stratified analysis of the events shows that the cardio-protective effect of DFP and Combination, compared to DFO, persists at all levels of haemosiderosis, as assessed by ferritin levels. This analysis was applied only to assess whether there was an advantage of the DFP and Combination compared to DFO and whether this advantage was the same at all levels of haemosiderosis. Although the reduced power because of stratification did yield statistically significant differences in only some comparisons, the trends seen are of particular interest. Even patients with mild haemosiderosis on DFO experience higher risk of cardiac disease compared to those on the other two regimes. This protective effect is more prominent with Combination. This indicates that patients who until recently were considered safe from developing cardiac disease have the potential to reduce their cardiac risk even further with the appropriate choice of chelation regime. A striking result, with respect to DFP, is that the protective effect reaches its maximum in patients with severe iron load and appears to be equal to that of combination therapy.

The applied criteria to the diagnosis of a cardiac event and its attribution to the chelation regime are similar to those in other studies (10, 11). It is clear that the regimes that became available after the year 2000 have had a significant impact on the reduction of cardiac morbidity. This is compatible with findings in prospective studies with DFO, DFP (16), Combination (19) and DFX (20) that assessed the clearance of iron from the heart based on CMR studies. Between the DFP and Combination, we could not discern any statistically significant difference in the risk of *de novo* cardiac events. A number of studies have shown that irrespective of acceptable chelation with DFO as demonstrated by mean ferritin levels, the incidence of cardiac morbidity remained a clinical issue. In some patients, even low levels of ferritin were associated with excess cardiac iron as assessed by CMR (21, 22). These recent studies have further demonstrated that both ferritin levels and LIC have no clinical relevance with respect to their ability to predict the degree of cardiac iron loading.

The observations of significance with respect to level of haemosiderosis, gender and age as confounding factors with respect to cardiac morbidity indicate that studies based on clinical observations which lack randomisation, should take these three parameters into account so that the results will be accurate.

The patients on DFP and Combination showed a significantly greater probability of reversing their cardiac dysfunction compared to DFO. Some patients showed an improvement in their cardiac function while remaining on DFO. It is well known and reported that intensification of DFO therapy is capable of reversing even overt cardiac failure (23–25). The ability to improve cardiac function with DFP (16) and Combination (26) are as expected and are compatible with other studies, clinical experience and case reports (27–29). The reticence of the cardiologists to cease the medications once there was a sustained improvement in cardiac function could have lead to an underestimation of the numbers of patients who showed improvement. Our intention was not to include patients as improved, even with possible minor residual cardiac dysfunction. Therefore, in the presence of overt cardiac failure, the preferred initial chelation regime should be Combination or intensive DFO therapy if DFP treatment is contraindicated.

The estimated low rate of *de novo* cardiac events and the absence of cardiac-related deaths, in the DFX group in our Unit are encouraging and to date have not been reported. They need, however, to be evaluated in the context of the features of the patients receiving DFX in our unit. As mentioned earlier, the medication was preferentially prescribed to patients who had mild iron load as assessed by ferritin levels and CMR (30). Further studies that will follow patients over a long period of time, may confirm the present preliminary results. The relatively short follow-up period in patients on DFX does not provide sufficient information to make appropriate inferences in the improvement component.

A major limitation in our study as with any clinical observational study is that treatment is modified according to the patients’ progress. This translates to situations where therapeutic decisions are made according to signs of deterioration and perceived risks. In our unit, patients who were at increased risk of cardiac disease based on CMR or ferritin levels are selectively prescribed Combination or intensive DFO. We believe that differences in the level of iron load which are related to the clinical decisions and could confound the results have been taken into account by the stratification analysis. With respect to the assignment criteria as to which regime the patient was taking at the time of an event, there were two patients in whom the event was attributed to the previous regime; not the one they were taking at the time of the diagnosis of the event. They were only taking the new regime for a brief period of time (35 and 73 d). It is likely that the regime changes were made on the basis of clinical concerns with respect to potential imminent cardiac disease. However, until the change in regime, they did not fulfil the criteria for characterisation as events. When the events were attributed to the regime received at the time of the event, the results showed minor differences without altering the clinical significance. As this was a clinical observational study, we did not record compliance or doses of chelation medication, as it is logistically impossible to calculate the average dose for a long period of time in which several minor changes could have been made. We consider the difficulties associated with the use of any prescribed regime as a parameter of its efficacy. Therefore, poor efficacy is related to possible poor compliance. This in fact, could be interpreted as a strong point of this study. In a controlled clinical trial, compliance is rigidly evaluated. The outcome reported is what might be expected within a clinical context and may be more valuable with respect to what a clinician can expect.

As in our experience, myocarditis usually reverses within a relatively short period of time. In some cases, it is associated with previous viral infections but the diagnosis can only be made for certain by cardiac biopsy or on necropsy. It was excluded as a cause of the cardiac event on the basis of the long period of time needed for reversal of cardiac dysfunction. For cardiac deaths, without necropsy analysis we cannot be sure that the deaths were not myocarditis.

We believe that the volume of data on DFP and Combination was satisfactory, albeit less, than that of DFO. This could have been a potential limitation in terms of reduced power only, because incorporation of age, gender and level of haemosiderosis in the analysis removed any confounding effect. Had the results not been statistically significant, we would have not been able to determine whether the lack of significance in the differences was related to the low power or that there were practically no differences between regimes.

## Conclusion

New chelation options in patients with thalassaemia have reduced cardiac morbidity and mortality. Cardiac deaths have reduced in many centres throughout the world since the year 2000. The newer chelation regimes demonstrate lower risk of cardiac events. In the past, the onset of cardiac failure in patients with thalassaemia usually progressed to death within a short period of time. This study clearly demonstrates that a significant proportion of patients who developed cardiac events, were able to regain normal cardiac function with appropriate therapy. The new regimes together with better non-invasive diagnostic facilities for identifying both hepatic and cardiac iron load are expected to improve survival even further and reduce incidence and severity of complications caused by haemosiderosis.
